# Hydroxytyrosol Improves Obesity and Insulin Resistance by Modulating Gut Microbiota in High-Fat Diet-Induced Obese Mice

**DOI:** 10.3389/fmicb.2019.00390

**Published:** 2019-03-04

**Authors:** Zhuoqun Liu, Ningning Wang, Yanan Ma, Deliang Wen

**Affiliations:** ^1^School of Public Health, Dalian Medical University, Dalian, China; ^2^School of Public Health, China Medical University, Shenyang, China; ^3^The Institute of Health Science, China Medical University, Shenyang, China

**Keywords:** hydroxytyrosol, fecal microbiota transplantation, obesity, inflammation, insulin resistance, liver steatosis

## Abstract

Obesity is a common chronic metabolic disease that is harmful to human health and predisposes the affected individuals to a cluster of pathologies. Insulin resistance (IR) is one of the most frequent complications of obesity. Hydroxytyrosol (HT) may reduce obesity and IR in high-fat diet (HFD)-fed mice; however, the mechanism underlying is still unknown. Systemic low-grade inflammation and intestinal dysfunction are thought to be associated with obesity and IR. In this study, we found that HFD feeding for 8 weeks altered the intestinal microbiota, injured intestinal barrier function, increased endotoxin release into the blood, enhanced the expression of inflammatory factors (TNF-α, IL-1β, IL-6) and lipid accumulation in liver, caused obesity, and aggravated IR via the JNK/IRS (Ser 307) pathway in HFD mice. We also found that HT gavage could reverse those effects and the beneficial effects of HT were transferable through fecal microbiota transplantation. Our data indicate that HT can improve obesity and IR by altering the composition of the intestinal microbiota and improving integrity of the intestinal wall. We propose that HT replenishment may be used as a dietary intervention strategy to prevent obesity and IR.

## Introduction

Childhood obesity is a major public health problem and is becoming increasingly prevalent in most regions of the world. The worldwide prevalence of childhood obesity has risen from 4% in 1975 to 18% in 2016, and recent studies have shown that more than 340 million children and adolescents are overweight or obese (Orlando et al., 2018). Obesity is often accompanied by chronic low-grade inflammation, disorders of glucose and lipid homeostasis, and metabolic diseases such as type 2 diabetes, cardiovascular disease, and hepatic steatosis (Hotamisligil, 2017). Childhood obesity may increase the likelihood of obesity and metabolic disorders in adulthood, greatly decreasing the individual’s quality of life (Bass and Eneli, 2015).

It is widely accepted that unhealthy diets, sedentary lifestyles, and sleep deprivation are associated with an increased risk of obesity (Baranowski and Taveras, 2018). In addition, a growing number of studies have shown that changes in gut microbiota in early life may also play a role in the development of obesity. There are trillions of microorganisms in the human body (Tanaka et al., 2009; Dominguez-Bello et al., 2010), which undergo dynamic changes during an individual’s life. Environmental factors (Zhang et al., 2010), including diets (Siddharth et al., 2013), are the main contributors to the composition of microorganisms rather than genetic factors. Most studies have shown that the fetus’s intestinal tract is sterile during pregnancy (Makino et al., 2013), and the initial bacterial colonization occurs during birth. It has been suggested that the methods of birth and feeding determine the type of bacteria which are colonized into the baby’s gastrointestinal tract (Munyaka et al., 2014). Moreover, the timing of supplementary food addition also affects the composition of the gut microbiota, after which the gut microbiota remains stable until old age (Villanueva-Millan et al., 2015).

The “Mediterranean diet” (MD), a diet rich in fruits and vegetables, fish, whole grains, beans, and olive oil, is considered to be one of the healthiest diets (Del Chierico et al., 2014). Numerous studies have shown that people in the Mediterranean region are far less likely to suffer from cardiovascular disease, diabetes, colon cancer, and rectal cancer than people in other European and American countries. Studies have shown that the MD increases *Bacteroides* and *Clostridium*, reduces *Proteus* and *Bacillus* populations in the gut microbiota (Marlow et al., 2013). A recent study showed that the MD could increase the gut microbiota diversity in cynomolgus monkeys (Nagpal et al., 2018), increase the abundance of *Lactobacillus* and *Clostridium*, and reduce the abundance of *Ruminococcus* and *Coprococcus*. The current literature on the role of the MD in childhood is lacking. Olive oil, an important component of the MD, is beneficial due to its abundance of monounsaturated fatty acids and antioxidants (Hamden et al., 2009). HT, the main component of olive oil, has been shown to have no toxic effects on cells and animals within a certain dose range (Auñon-Calles et al., 2013a,b; D’Angelo et al., 2001). Previous experiments have shown that HT prevents obesity, inflammation, hyperglycemia, and IR induced by a HFD (Carito et al., 2013; Cao et al., 2014; Voigt et al., 2015; Wang et al., 2018). HT may decrease lipid deposits by downregulating the SREBP-1c/FAS pathway in the liver and skeletal muscle tissues in HFD-fed mice (Cao et al., 2014). Additionally, HT regulates Toll-like receptor 4 (TLR-4)-dependent inflammation through a NF-κB-independent pathway to relieve oxidative stress (Takeda et al., 2014). Our recent research has revealed that HT improves inflammation, IR, and hepatic steatosis by reducing endoplasmic reticulum (ER) stress and by regulating the JNK/IRS pathway in HFD-induced obese mice (Wang et al., 2018). However, little research has been done on the effect of HT on the gut microbiota. In the present study, we hypothesized that HT may prevent obesity and IR by altering the composition of the intestinal microbes and alleviating inflammatory status. Therefore, we examined the effect of HT supplementation on HFD-induced obese mice in early life and explored the mechanism of action of the gut microorganisms through bacterial fecal transplantation experiments.

## Materials and Methods

### Animal and Dietary Intervention

Twenty-eight three-week-old male C57BL/6J mice were raised at the Specific Pathogen Free Animal Experimental Center of Dalian Medical University (The Ethical approval number of C57BL/6J mice is AEE17057) and were divided into 4 groups: control group (chow), HFD group, HT group, and HTF. Seven mice per cage were housed in the following conditions: 12/12 hour light/dark cycle, maintained temperature of 23°C ± 2°C, and a humidity of 60%, with free access to food and water. The chow group was fed a normal diet as a reference, and the other three groups were fed an HFD (45% kcal fat content, MD12032, Medicience Ltd.). The intragastric interventions were performed for eight weeks as follows: the chow group and HFT group were gavaged with distilled water (10 ml/kg/day), the HT group was gavaged with HT (50 mg/kg/day, dissolved in distilled water, purity ≥ 98%, APP-ChemBio, China), the HTF group was subjected to fecal transplantation (fecal transplant donor from the HT group, 100 mg/1 mL sterile saline).

### Fecal Collection and Transplantation

One hundred milligrams of fresh feces (collected during the experiment every day) from the HT group were dissolved in 1 mL of sterile saline in an autoclaved tube, vortexed with a turbine for 10 s, and then centrifuged at a speed of 800 *g* for 3 min (Biofuge Primo R, Thermo). The prepared solution was used within 10 min as described previously (Chang et al., 2017).

### Oral Glucose Tolerance Tests and Insulin Tolerance Tests

After 8 weeks of feeding, the mice were fasted for 10 h overnight without restrictions on drinking water and gavaged with glucose solution [2.0 g/kg body weight (bw)] for OGTT. For ITT, the mice were fasted for 5 h and injected intraperitoneally with insulin (0.75 unit/kg bw, FosunPharm). Tail vein blood was collected, blood glucose was measured using a glucometer (Yue Zhun Type II 560, Yuyue), and the area under the curve (AUC) was calculated as described previously (Wang N. et al., 2017).

### Sample Collection and Preservation

After the mice were sacrificed, the liver, scWAT, BAT, rWAT, epididymal white adipose tissue (eWAT), and ileum were removed, weighed, and wrapped in aluminum foil. The blood samples were divided into two parts: one part was centrifuged (4°C, 3000 rpm, 15 min) for serum, and another part was centrifuged (4°C, 3000 rpm, 15 min) for plasma in a test tube containing heparin. All samples were placed in liquid nitrogen for quick freezing and stored at -80°C for further use.

### Hematoxylin and Eosin Staining

A portion of the scWAT was fixed in 10% formalin overnight and was then dehydrated and embedded in paraffin. Sections (thickness of 4-5 μm) were stained with hematoxylin and eosin and were then visualized with a microscope (Olympus BX72, Japan). Cell size was analyzed using Image J software (National Institutes of Health).

### Oil Red O Staining

The liver tissue was successively placed in 10, 20, and 30% sucrose solutions for gradient dehydration. The dehydrated liver was processed into frozen sections (thickness of 6 μm), stained with oil red (WLA055a, Wanleibio) for 10 min, and observed using a microscope. The size of the lipid droplets was analyzed using Image J software.

### Biochemical Analysis

Plasma lipopolysaccharide (LPS) levels were quantified using a Chromogenic Endpoint Tachypleus Amebocyte Lysate (CE TAL, Xiamen Bioendo Technology Co., Ltd., China) assay according to the manufacturer’s instructions. The concentrations of TNF-α, IL-1β, and IL-6 in liver were analyzed using commercial kit (MB-2868A, MB-2776A, MB-5737A, Meibiao, China). Fasting serum insulin concentrations were determined using a commercial ELISA kit (ER1113, FineTest, China). IR was assessed using the index of HOMA-IR: fasting blood glucose (mmol/L) × fasting blood insulin (mU/L) / 22.5.

### Gut Microbiota Analysis

Before the mice were sacrificed, stool samples were collected for gut microbiota analysis using an autoclaved tube. Fecal DNA was extracted from the stool samples using the E.Z.N.A Soil DNA Kit (Omega Bio-tek, United States) according to the manufacturer’s protocol. The hypervariant region V3–V4 of the bacterial 16S rRNA gene was amplified with the primers 338F (5′-ACTCCTACGGGAGGCAGCA-3′) and 806R (5′-ACTCCTACGGGAGGCAGCA-3′) by PCR. The PCR product was recovered using a 2% agarose gel, purified using an AxyPrep DNA Gel Extraction Kit (Axygen Biosciences, United States), eluted with Tris-HCl, and detected by 2% agarose electrophoresis. Quantification detection was performed using Quantifluor-ST (Promega, United States). Sequencing was performed using Illumina’s MiSeq PE300 platform (Shanghai Meiji Biomedical Technology Co., Ltd., China). UPARSE software was used to perform operational taxonomic unit (OTU) clustering of sequences based on 97% similarity (version 7.1^1^). The OTU was subsampled for further analysis. Chimeras were eliminated using UCHIME software. Each sequence was compared to the Silva database (SSU123) for species classification annotation using the RDP classifier^2^, with the alignment threshold set to 70%. The Wilcoxon rank-sum test was used to compare differences between the two groups.

The R language tool was used to make the graph of the Rank-Abundance curve, the Venn diagram, and the community histogram/heatmap. Rarefaction curve and alpha-diversity indexes were analyzed by Mothur. Sample hierarchical clustering and principal coordinates analysis (PCoA) were performed using the Bray-Curtis distance algorithm, and the graphics were created with R language. Community comparison was evaluated using the UniFrac Server followed by a Wilcoxon rank sum-test.

### Western Blotting

Total proteins were extracted from the liver and ileum using total protein extraction kits (KGP1100, KeyGEN BioTECH). The concentration of the extracted proteins was quantified using a Pierce BCA Protein Assay Kit (Thermo). Equal amounts of protein from each sample were transferred to a polyvinylidene fluoride membrane after polyacrylamide gel electrophoresis. After blocking with 10% skim milk for 1 h at 37°C in a water bath, the blot was incubated with primary antibody (a suitable concentration diluted with TBST) overnight at 4°C. After being washed with TBST (5 times, 5 min at a time), the blot was incubated with secondary antibody for 2 h at room temperature. We used a Bio-Rad ChemiDoc MP imaging system to detect the bands of target proteins, and the relative density of the bands was analyzed using Image J software.

### Antibodies

Primary antibodies: internal control β-actin (1:1000; TA-09, ZSGB-BIO), GAPDH (1:1000; 10494–1-AP, Proteintech), IL-1β (1:1000; WL00896, WanleiBio), IL-6 (1:1000; WL01678, WanleiBio), TNF-α (1:1000; WL02770, WanleiBio), JNK (1:500; WL01295, WanleiBio), phosphorylated JNK (Thr183 Tyr185) (1:500; WL01813,WanleiBio), AKT (1:1000; 10176-2-AP, Proteintech), phosphorylated AKT (Ser473) (1:500; WLP001a, WanleiBio), TLR4 (1:500; WL00196, WanleiBio), Tight junction protein 1 (ZO-1) (1:500; WL03419, WanleiBio), Occludin (1:1000; WL01996, WanleiBio), IRS-1 (insulin receptor substrate-1, 1:1000; #2382, CST), phosphorylated IRS-1 (serine 307, 1:500; WH081658, ABClone). Secondary antibodies: peroxidase-conjugated goat anti-mouse IgG (1:5000; ZB-2305, ZSGB-BIO) or peroxidase-conjugated goat anti-rabbit IgG (1:5000; ZB-2301, ZSGB-BIO).

### Statistical Analysis

The data are expressed as the means ± standard error of mean (SEM) and were analyzed and plotted with GraphPad Prism 6 (GraphPad Software, United States). Data sets that involved more than two groups were assessed by one-way ANOVA followed by Tukey’s honest significant difference *post hoc* tests. *P*-value ≤ 0.05 was considered statistically significant.

## Results

### HT and HTF Reversed HFD-Induced Obesity in Mice

**Figure 1:**
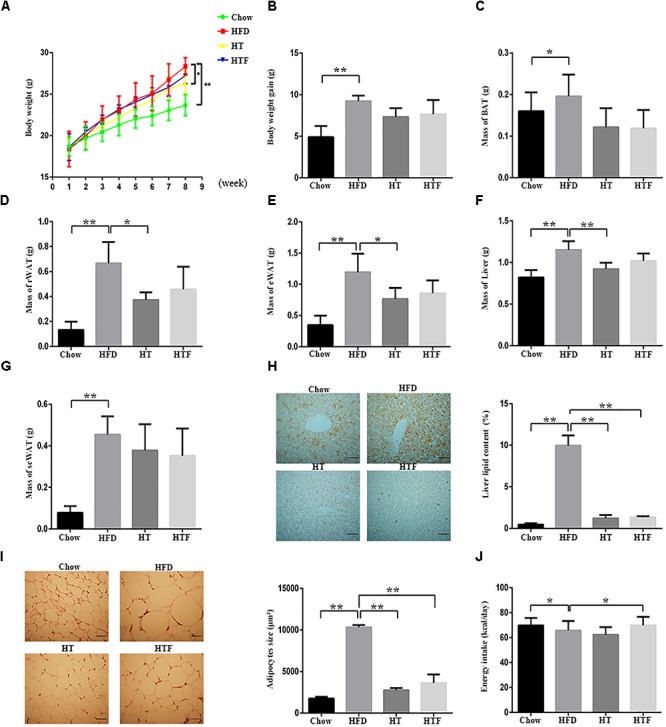
HT and HTF reduces body weight and fat accumulation in HFD-fed mice. HFD-fed mice were treated daily with HT (50 mg/ kg) or HTF (100 mg/1 ml) by intragastric gavage for 8 weeks (*n* = 7 for each group). Effects of HT and HTF treatment on body weight **(A)** body weight gain **(B)** brown fat **(C)** perirenal fat **(D)** epididymal fat **(E)** liver weight **(F)** subcutaneous fat **(G)** oil red O staining **(H)** subcutaneous adipocyte size **(I)** and energy intake **(J)**, are shown. In **(H)**, Liver lipid content was assessed using the Image J software. Scale bar, 50 μm. In **(I)**, adipocyte size was estimated using the Image J software (lower panel). Scale bar, 50 μm. Graph bars with statistically significant results (*P* ≤ 0.05) based on one-way ANOVA analysis followed by Tukey’s honest significant difference *post hoc* tests. Data are shown as mean ± s.e.m (^∗^*P* ≤ 0.05, ^∗∗^*P* ≤ 0.01).

To determine whether HT supplementation or fecal transplant from HT-supplemented mice would reduce obesity in HFD mice, we recorded the body weights of the mice and the excised adipose tissue. HFD induced significant increases in final body weight, body weight gain, liver weight, and tissue mass of the BAT, perirenal adipose tissue (rWAT), epididymal white adipose tissue (eWAT), and the scWAT compared with the chow group. HT supplementation significantly reduced the final body weight, mass of the rWAT, eWAT mass, and liver mass compared with the HFD group. The body weight gain and the weights of the BAT and scWAT tended to decrease, but there were no statistically significant differences (Figure 1A–G). All of the above parameters also declined in the HFT group compared to the HFD group, but none reached significance (Figure 1A–G). Consistently, we observed evident lipid deposition in the hepatocytes and increases in adipocyte size in the HFD group compared to the chow group, which were reversed by HT supplementation and HFT (Figure 1H–I). Interestingly, HFD mice consumed less energy than the chow group, and the HFT mice consumed more energy than the HFD mice (Figure 1J).

### HT and HTF Promoted Intestinal Integrity and Alleviated Inflammation in HFD Mice

**Figure 2:**
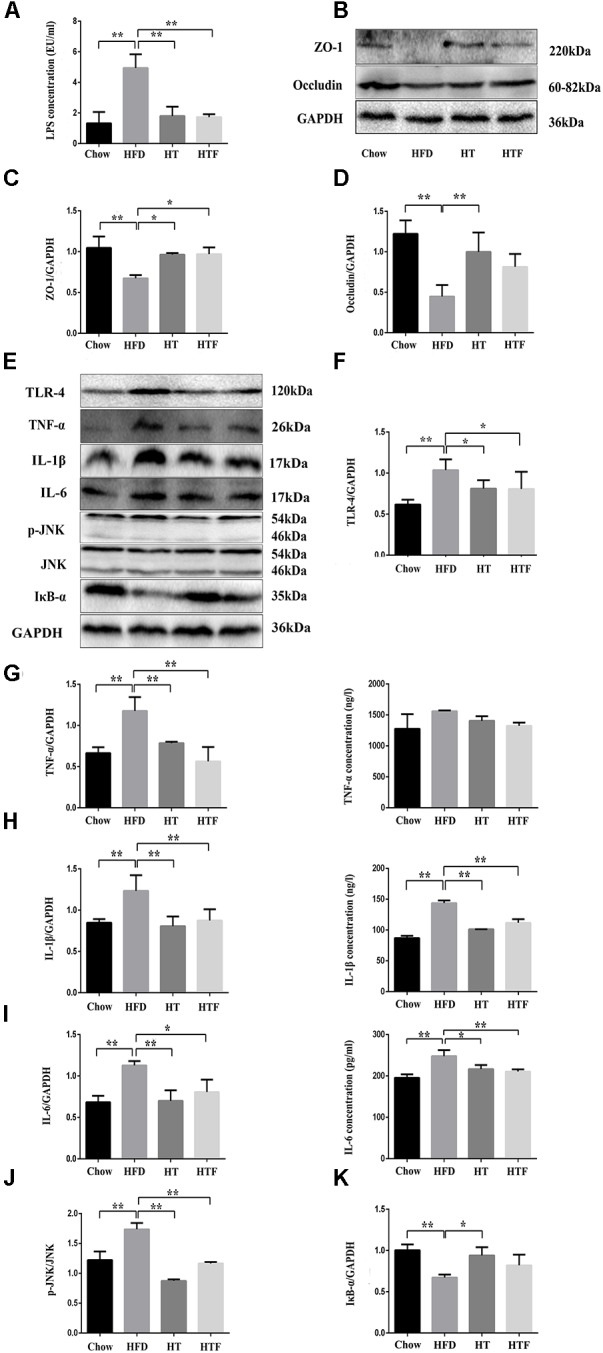
HT and HTF protected intestinal integrity, decreased concentration of plasma LPS and alleviated inflammation in HFD mice. Effects of HT and HTF treatment on plasma endotoxin using a Chromogenic End-point Tachypleus Amebocyte Lysate **(A)** The expression of Occludin, ZO-1 and GAPDH in ileum was assessed using western blot **(B)**. Bar graphs represent normalized data of Occludin /GAPDH **(C)** and ZO-1 /GAPDH **(D)** *n* = 3. The expression of Occludin, TLR-4, TNF-α, IL-1β, IL-6, p-JNK, IκB-α and GAPDH in liver was assessed using western blot **(E)**. Bar graphs represent normalized data of TLR-4/GAPDH **(F)**, TNF-α/ GAPDH and the concentration of TNF-α in liver **(G)**, IL-1β/GAPDH and the concentration of IL-1β in live **(H)**, IL-6/GAPDH and the concentration of IL-6 in live **(I)**, p-JNK/JNK **(J)**, IκB-α/GAPDH **(K)** *n* = 3. Molecular weight markers were indicated as kilodaltons (kDa), Graph bars with statistically significant results (*P* ≤ 0.05) based on one-way ANOVA analysis followed by Tukey’s honest significant difference *post hoc* tests. Data are shown as mean ± s.e.m (^∗^*P* ≤ 0.05, ^∗∗^*P* ≤ 0.01).

To examine whether HT and HTF can improve intestinal integrity, we measured the concentration of lipopolysaccharide (LPS) in the plasma and the protein expressions of ZO-1 and occludin in the ileum. As shown in Figure 2A–D, HFD increased the plasma LPS levels and disrupted the intestinal barrier, whereas HT and HTF were able to reduce the plasma LPS concentration and prevent intestinal barrier damage. Thus, HT and HTF may reduce the release of LPS into the blood by promoting intestinal barrier integrity. Additionally, intestinal integrity can also affect inflammation levels. As expected, the protein expressions of TLR-4, TNF-α, IL-1β, IL-6, and p-JNK in the mouse livers increased significantly in the HFD group compared to the chow group (Figure 2E–J). We further discovered that the concentration of the liver’s IL-1β and IL-6 declined significantly after HT and HTF intervention, the concentration of TNF-α have only a downward trend (Figure 2G–I). Simultaneously, the expression of IκB-α, significantly decreased compared to the chow group (Figure 2K). The expression of TLR-4, TNF-α, IL-1β, IL-6, and p-JNK were significantly decreased in the HT and HTF groups (Figure 2E–J). The expression of IκB-α was significantly decreased in the HT group, but did not change significantly in HTF group (Figure 2K). Previous studies have shown that the TLR4 signaling pathway promotes the production of pro-inflammatory cytokines through the activation of JNK and NF-κB. Increased phosphorylation of JNK and NF-κB ultimately triggers IR via phosphorylation of IRS-1 on serine-307 and deactivation of AKT.

### HT and HTF Decreased IR in HFD-Fed Mice

**Figure 3:**
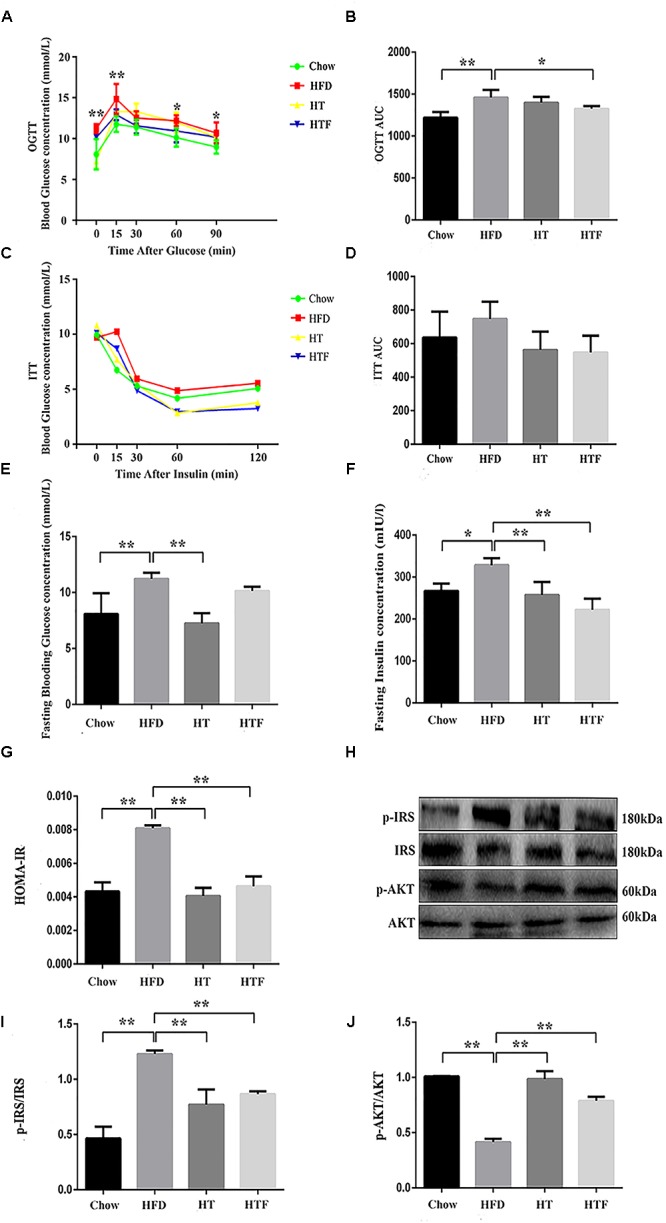
HT and HTF improved IR in HFD-fed mice. Oral Glucose tolerance test (OGTT, 2 g/kg bw) after 8 weeks of intervention ^∗^*P* < 0.05, ^∗∗^*P* < 0.01 HFD vs. Chow **(A)** and the area under the curve (AUC) calculation **(B)**, Insulin tolerance test (ITT, 0.75 U/kg bw) after 8 weeks of intervention **(C)** and the AUC calculation **(D)**, Fasting blood glucose levels of each group **(E)**, Fasting blood insulin of each group **(F)** and HOMA-IR index after 8 weeks of intervention **(G)**. The expression of p-IRS and p-AKT in liver was assessed using western blot **(H)**. Bar graphs represent normalized data of p-IRS/IRS **(I)** and p-AKT/AKT **(J)** *n* = 3. Molecular weight markers were indicated as kilodaltons (kDa), Graph bars with statistically significant results (*P* ≤ 0.05) based on one-way ANOVA analysis followed by Tukey’s honest significant difference *post hoc* tests. Data are shown as mean ± s.e.m (^∗^*P* ≤ 0.05, ^∗∗^*P* ≤ 0.01). HOMA-IR: homeostasis model assessment of insulin resistance.

To evaluate insulin sensitivity, we performed oral glucose tolerance tests (OGTT) and insulin tolerance tests (ITT), tested fasting blood glucose and insulin, and calculated the index of HOMA-IR (Figure 3A–G). Specifically, after 8 weeks of a HFD, the OGTT area under the curve (OGGT-AUC) (Figure 3A), fasting blood glucose concentration (Figure 3E), fasting insulin concentration (Figure 3F), and HOMA-IR index (Figure 3G) were enhanced significantly in the HFD group compared to the chow group. In addition, the ITT-AUC (Figure 3D) tended to increase. The fasting blood glucose concentration, fasting insulin concentration, and HOMA-IR index were decreased significantly in HT group; and the OGTT-AUC, fasting insulin concentration, and HOMA-IR index were decreased significantly in HTF group. Although the other indicators also decreased with HT and HTF, no significant differences were found. These results indicate that HT supplementation and HTF alleviated IR induced by HFD. Furthermore, we measured the expression of key proteins related to IR (Figure 3H). As shown in Figure 3I–J, after 8 weeks of HFD, the expression of p-IRS (Figure 3I) was elevated significantly compared to the chow group, and the expression of p-AKT (Figure 3J) was significantly decreased. The expression of p-IRS and p-AKT (Figure 3I–J) significantly decreased in HT and HTF groups.

### HT and HTF Modulated Gut Microbiota Composition and Reversed HFD-Induced Gut Dysbiosis

To explore changes to the gut microbiota after HFD, HT, and HTF, we performed 16S rRNA gene analysis of fecal samples from the mice. High-throughput sequencing yielded 1574700^∗^2 original sequences from 24 samples (Supplementary Table S1), of which 1,090,437 were valid sequences (33,690 ± 4,147 per sample) (Supplementary Table S2). The coverage index was 1 ± 0.00001, indicating adequate species coverage during sequencing (Supplementary Table S3).

**Figure 4:**
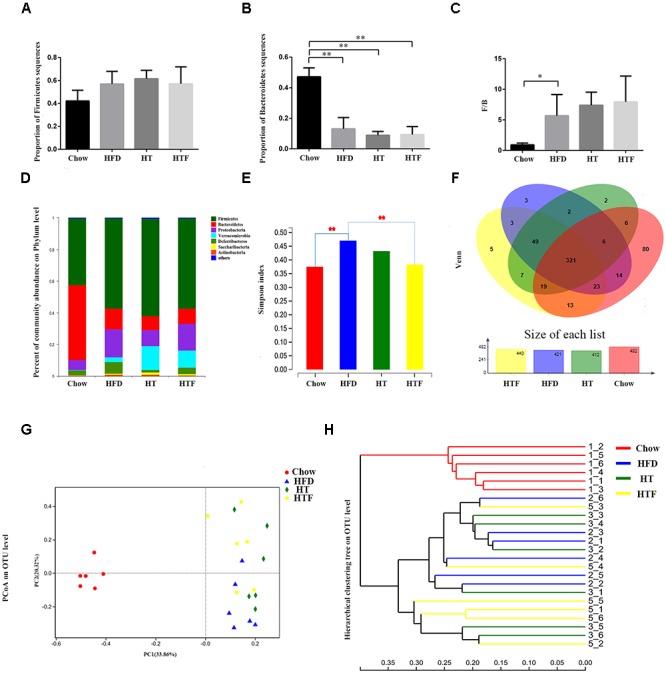
HT and HTF alters microbiota structure in HFD-fed mice. Microbiota composition in feces of chow-fed mice and HFD mice treated with HT and HTF were analyzed using next generation sequencing (*n* = 6 for each group). Proportion of Firmicutes **(A)**, Proportion of Bacteroidetes **(B)**, F/B **(C)**, Graph bars with statistically significant results (*P* ≤ 0.05) based on one-way ANOVA analysis followed by Tukey’s honest significant difference *post hoc* tests. Plots shown were generated using the weighted version of the bray curtis-based Bacterial taxonomic profiling in the phylum level of intestinal bacteria from each group **(D)** Simpson index **(E)** were analyzed using unpaired two-tailed Student’s *t*-test. Venn chart **(F)**, PCoA **(G)** and Hierarchical clustering tree on OTU level **(H)** (^∗^*P* ≤ 0.05; ^∗∗^*P* ≤ 0.01).

We found no significant differences in *Firmicutes* between the four groups (Figure 4A). However, the number of *Bacteroides* in the chow group was significantly higher than the HFD-fed groups (Figure 4B). The Chow group had the lowest ratio of *Firmicutes* to *Bacteroides* (F/B) compared to the other three groups (Figure 4C). Neither the number of *Bacteroides* nor the ratio of F/B were altered after HFD, HT, and HTF (Figure 4B–C). Notably, taxonomic profiling showed that supplementation with HT and HTF reduced the numbers of *Proteobacteria* and *Deferribacteres* in HFD-fed mice (Figure 4D).

The Simpson Index was proposed by Edward Hugh Simpson in 1949 to quantify the biodiversity of a region and serves as an indicator of microbial diversity. Larger Simpson Index values indicate lower diversity in the community. The Simpson index of HFD group was significantly higher than that of the chow group and was decreased after HT and HTF treatment, albeit no statistical significance was observed in the HT group (Figure 4E).

All sequences were classified into 553 operational taxonomic units (OTUs) at 97% similarity after subsampling. The Venn diagrams in Figure 4 showed that the OTU of the chow group was the highest, reaching 482, followed by 440 in the HTF group, 421 in the HFD group, and 412 in the HT group. 553 OTUs were found in all of the groups. In this study, there were 80 OTUs unique to the chow group, 3 OTUs unique to the HFD group, 2 OTUs unique to the HT group, and 5 OTUs unique to the HFT group (Figure 4F).

We used principal coordinates analysis (PCoA) plots (Figure 4G) and sample-level clustering analysis (Figure 4H) to reflect the similarities and differences in the composition of the microbial community. In the PCoA plots, the chow group is distributed on the left side, the HFD group is mostly distributed in the lower right corner, the HTF group is mostly distributed in the upper right corner, and the HT group is evenly distributed on the right side. The results showed a clear difference in the gut microbiota composition between the chow group and the other three groups, and the microbiota composition of HT and HTF groups were similar.

**Figure 5:**
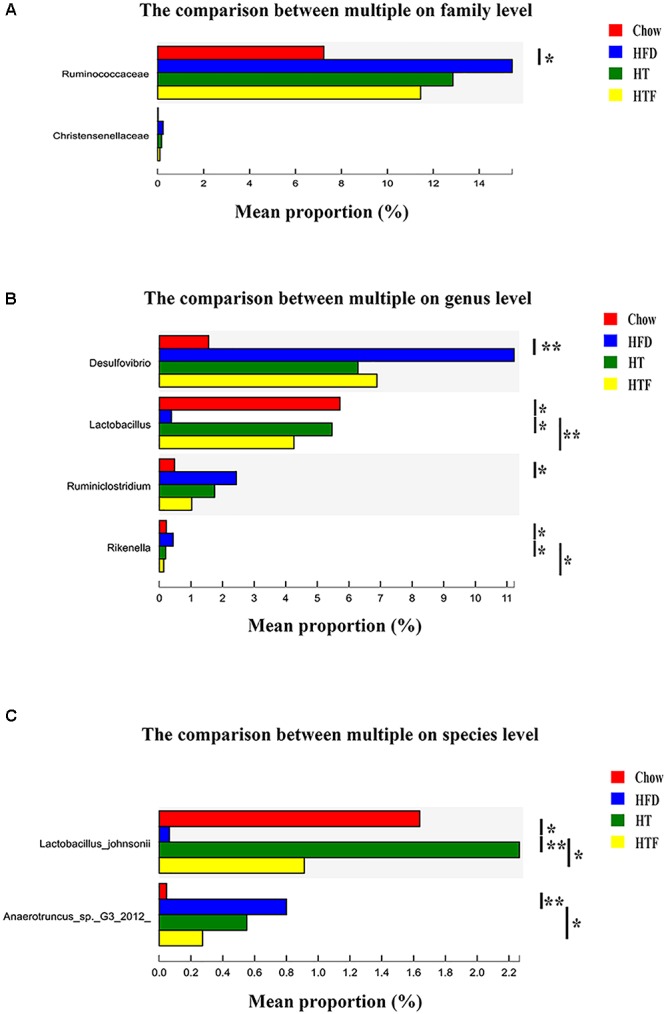
The analysis of species difference in different groups. The comparison between HFD and the other three groups in family **(A)**, genus **(B)** and species **(C)** level based on wilcoxon rank sum test (^∗^*P* ≤ 0.05; ^∗∗^*P* ≤ 0.01).

To further explore bacterial species changes in obese mice undergoing HT or HTF treatment, we performed a significant difference comparison between the groups. Since a large number of bacteria were found in this study, statistical differences at the species, genus and family levels were calculated. At the species level, we found that 2 species were altered by HT and/or HTF. *Lactobacillus johnsonii* was significantly increased after HT supplementation and HTF, while *Anaerotruncus sp. G3 (2012) was* significantly decreased after HTF supplementation (Figure 5A). At the genus level, four representative genera were altered by HT and/or HTF treatment. *Lactobacillus* significantly increased after HT and HTF, while *Rikenella* was significantly decreased after HT and HTF. *Desulfovibrio* and *Ruminiclostridium* were decreased after HT and HTF, but the decreases were not statistically significant (Figure 5B). At the family level, *Ruminococcueae* and *Christensenellaceae* were decreased after HT and HTF, but the decreases were not statistically significant (Figure 5C).

## Discussion

Previous studies have shown that HT prevents inflammation, hyperglycemia, hyperlipidemia, IR, and obesity. Some studies have shown that HT supplementation is beneficial to HFD-induced obese mice, while other studies have shown that HT has no effect on obesity (Cao et al., 2014; Voigt et al., 2015; Wang et al., 2018). Specifically, Voigt et al. (2015) found that HT (20 mg/kg/day, 21 days) reduced weight gain in HFD mice, but there have statistical difference only on the 15th and 18th days. A previous study has showed (Jemai et al., 2015) that HT treatment (8 mg/kg, 16 mg/kg, 4 weeks) has no effect on the body weight of diabetic rats, but can improve plasma glucose, liver glycogen and total cholesterol levels. At the same time, high doses significantly improved plasma glucose and total cholesterol levels compared to low doses. In addition, Zheng et al. (2015) found that the neurons factor of the cerebral cortex were lost in db/db mice compared with the control mice. HT supplements (10 mg/kg, 50 mg/kg, 8 weeks) improved the neuronal survival of brain in db/db mice. Of note, high doses significantly improved neuronal survival compared to the low doses. The differences in these results may be due to a difference in HT concentration and intervention time. In the present study, HT gavage at a concentration of 50 mg/kg/day for 8 weeks was able to maintain the integrity of intestinal barrier, alter the composition of gut microbiota, reduce the release of endotoxin into the blood, and reduce inflammation, ultimately preventing HFD-induced obesity and IR.

Jumpertz et al. (2011) found that the gut microbiota is associated with energy metabolism (Bäckhed et al., 2004; Fleissner et al., 2010; Velagapudi et al., 2010). Our results demonstrated that mice fed regular chow consumed more energy than HFD-fed mice, but the body weight of the chow group was lower than that of the HFD mice, indicating that HFD induces body weight increase by regulating energy utilization. In addition, we observed that the body weight of the HTF mice was lower and the energy intake was higher than that of the HFD mice, suggesting that changes in the gut microbiota caused by HT may affect energy absorption and metabolism. Interestingly, the energy intake of the HT mice was lower than that of the HTF group, and more metabolic benefits were seen in the HT group. It is possible that the concentration used for the fecal transplantation was not enough, or that HT may benefit HFD mice through a mechanism which isn’t dependent upon gut microbiota.

Previous studies have highlighted that HFDs reduce microbial diversity (Turnbaugh et al., 2008; Turnbaugh et al., 2009). In contrast, Kasai et al. (2015) found that the bacterial diversity in obese individuals was significantly higher than that in lean individuals in clinical trials. We observed that HT and HTF restored bacterial diversity which had been decreased by HFD. Our results suggest that bacterial diversity is inversely proportional to obesity. Moreover, one of the characteristics of the gut microbiota in obese individuals is a high ratio of F/B (Ley et al., 2005; Ley et al., 2006; Turnbaugh et al., 2006). We found that HFD mice had a significantly increased F/B, which was not altered by HT or HTF intervention. This indicates that HT may be beneficial for metabolism but does not regulate F/B.

We found that HT supplementation significantly changed the composition of the gut microbiota. Consistent with the findings of Kim et al. (2012), we found that *Ruminococcaceae* were significantly decreased by HT supplementation at the family level. In addition, *Proteobacteria* and *Ferribacter* were increased in the HFD-fed mice, consistent with previous studies (Hu et al., 2015; Wang C. C. et al., 2017). The proportions of these two species decreased after HT intervention but did not reach statistical significance. This suggests that HT may prevent obesity partially through regulation of *Proteobacteria* and *Ferribacter*. In addition to these bacteria, other bacteria may also help to prevent obesity. It has been reported that oral administration of *Parabacteroides goldsteinii* reduced the body weight of HFD-induced obese mice, enhanced adipose tissue heat production, enhanced intestinal barrier integrity, and mitigated inflammation and IR (Wu et al., 2018). In the present study, we found that HT reversed the decrease of *Parabacteroides* after a HFD, suggesting that *Parabacteroides* may play an anti-obesity role. Moreover, clinical studies by Million et al. (2012) revealed that some *Lactobacillus* species were associated with normal body weight. We found that HT reversed the alteration of *Lactobacillus johnsonii* by HFD. Furthermore, *Christensenellaceae*, which belong to the *Firmicutes*, are enriched in low BMI individuals compared to those with a high-BMI (Goodrich et al., 2014) and are negatively correlated with obesity. Conversely, in our study, HT reversed the rise of *Christensenellaceae* caused by HFD. Finally, we found that HT reversed the decrease of *Rikenella* induced by HFD. HT enhanced a variety of bacterial species that are negatively associated with obesity and reduced species that are positively associated with obesity. In summary, HT may prevent HFD-induced obesity by regulating a variety of different gut microbiota.

The successes of FMT serve as direct evidence of the interaction between the gut microbiota and many diseases (Zhang et al., 2018). Previous studies have shown that FMT can affect body weight and some metabolic indicators. For example, Maria et al. found that fecal transplantation from chow-fed mice could increase gut microbiota species diversity and abundance in recipient HFD-fed mice without changing body weight (Kulecka et al., 2016). *Ridaura et al.* found that FMT recipients receiving a transplant from an obese individual gained more weight than those receiving a transplant from a non-obese individual (Ridaura et al., 2013). There is still a lack of research on the mechanism by which HT improves obesity. In order to investigate whether the gut microbiota played a role, we transplanted the fecal bacteria from mice fed HT to HFD mice. The PCoA and the hierarchical clustering tree indicate that the gut microbiota of the HT group and the HTF group were similar but not identical. This result reflects the effectiveness and reliability of the gut microbiota transplantation.

We found that FMT from mice supplemented with HT (HTF) also resulted in beneficial effects similar to those seen in HT-gavaged mice, including an increase in the integrity of the intestinal barrier, prevention of inflammation, and alleviation of obesity and IR induced by a HFD. Furthermore, HFT altered the intestinal microbiota in a manner consistent with HT supplementation. It is worth mentioning that most of the beneficial effects seen after HTF intervention were weaker than those seen after HT, which may be the result of an insufficient concentration of fecal bacteria. These results indicate that HT may benefit obese individuals by changing the composition of the intestinal flora.

In addition to analyzing changes in microbiota, our *in vivo* experiments showed that administration of HT reduced the expression of proteins involved in inflammation and reduced IR in HFD-fed mice. Our results suggest that a HFD induced intestinal dysfunction and increased the permeability of the intestinal mucosa in mice, which resulted in increased endotoxin release into the blood. Endotoxins trigger inflammation by activating the TLR-4 receptor and the NF-κB pathway, which induces IRS-1 phosphorylation at serine 307 and ultimately leads to IR and obesity (Wang N. et al., 2017). In the present study, HT and HTF restrained the activation of the TLR-4 and NF-κB pathways, reduced the expression of pro-inflammatory factors and IRS-1 phosphorylation at serine 307, and enhanced the phosphorylation of AKT. Thus, HT may alleviate IR through the TLR-4 and NF-κB signaling pathways.

There were a few limitations in our experiments. Although numerous bacteria related to the improvement of obesity have been identified, no further SMTs have been carried out. Additionally, our study was exclusively performed in animals. Future work should verify the effects of specific gut bacteria on obesity and IR, study the synergy of various gut bacteria by SMT, and explore the application of SMT in the treatment of obesity in humans. Overall, our results suggest that HT reduces weight gain, chronic inflammation, and IR in HFD-induced obese mice, partially through regulation of specific gut microbiota.

Overall, our results suggested that HT reduced weight gain, chronic inflammation and IR in HFD-induced obesity mice, partially by beneficial changes in specific gut microbiota.

## Data Availability

All datasets generated for this study are included in the manuscript and/or the Supplementary Files.

## Ethics Statement

This study was carried out in accordance with the recommendations of Animal Ethics Committee of the Institute of Genome Engineered Animal Models for Human Disease, Dalian Medical University. The protocol was approved by the Animal Ethics Committee of the Institute of Genome Engineered Animal Models for Human Disease, Dalian Medical University.

## Author Contributions

DW gave a general research direction. ZL completed the experimental design, data analysis, and manuscript writing. NW and YM conducted article language retouching. All authors reviewed the manuscript.

## Conflict of Interest Statement

The authors declare that the research was conducted in the absence of any commercial or financial relationships that could be construed as a potential conflict of interest.

## Footnotes

1 http://drive5.com/uparse/

2 http://rdp.cme.msu.edu/

## Supplementary Material

TABLE S1 Original sequences information.

TABLE S2 Valid sequences information.

TABLE S3 Coverage index.

## Abbreviations

BAT: brown adipose tissue
eWAT: epididymal white adipose tncipal coordinates analysis
FMT: fecal microbiota transplantation
HFD: high-fat diet
HOMA-IR: homeostasis model assessment of insulin resistance
HT: hydroxytyrosol
HTF: HT fecal microbiota transplantation group
IR: insulin resistance
MD: Mediterranean diet
rWAT: perirenal white adipose tissue
scWAT: subcutaneous white adipose tissue
SMT: selective fecal transplant experiment
