# Supplementary figures and images for: Hydroxytyrosol Improves Obesity and Insulin Resistance by Modulating Gut Microbiota in High-Fat Diet-Induced Obese Mice
Source: Front Microbiol. 2019 Mar 4;10:390. doi: 10.3389/fmicb.2019.00390 (PMC6410680; doi:10.3389/fmicb.2019.00390)

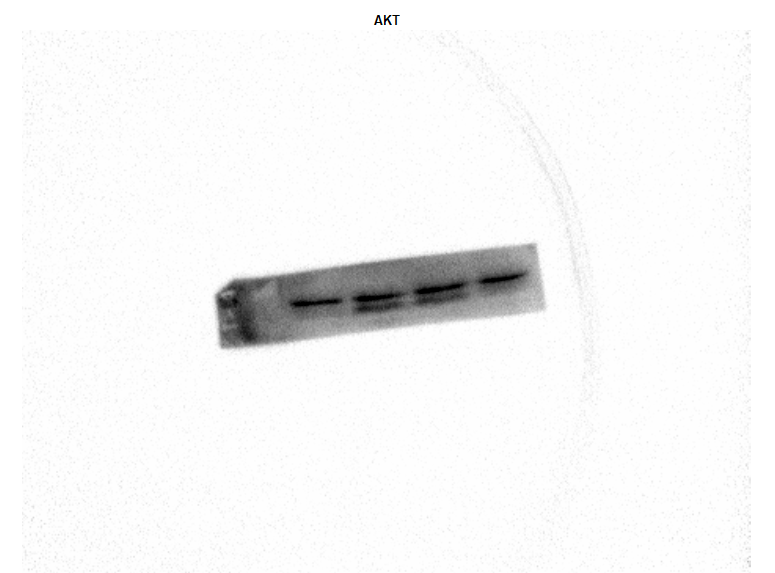

Supplement: Supplementary file 4 [file Data_Sheet_1.ZIP › AKT.tif]

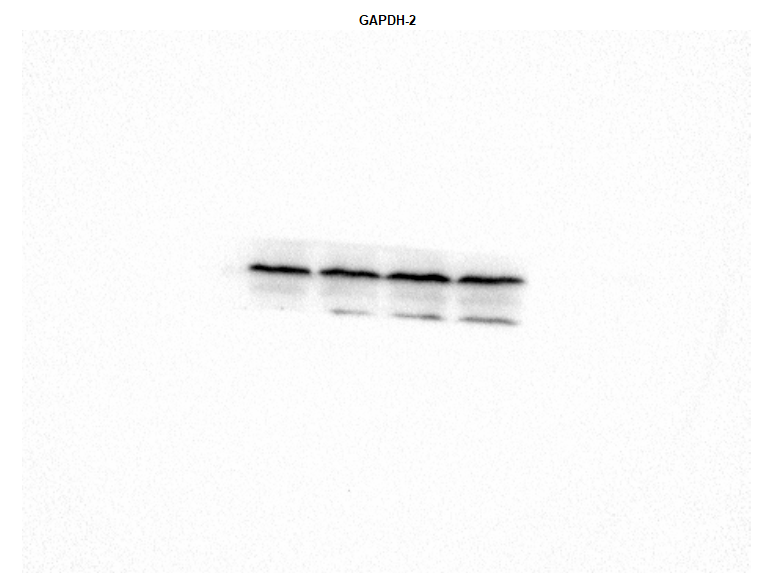

Supplement: Supplementary file 4 [file Data_Sheet_1.ZIP › GAPDH-2.tif]

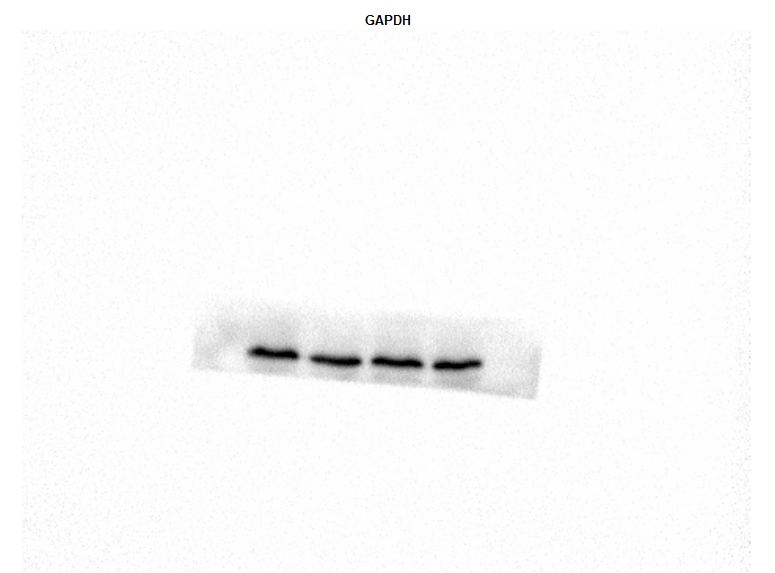

Supplement: Supplementary file 4 [file Data_Sheet_1.ZIP › GAPDH.tif]

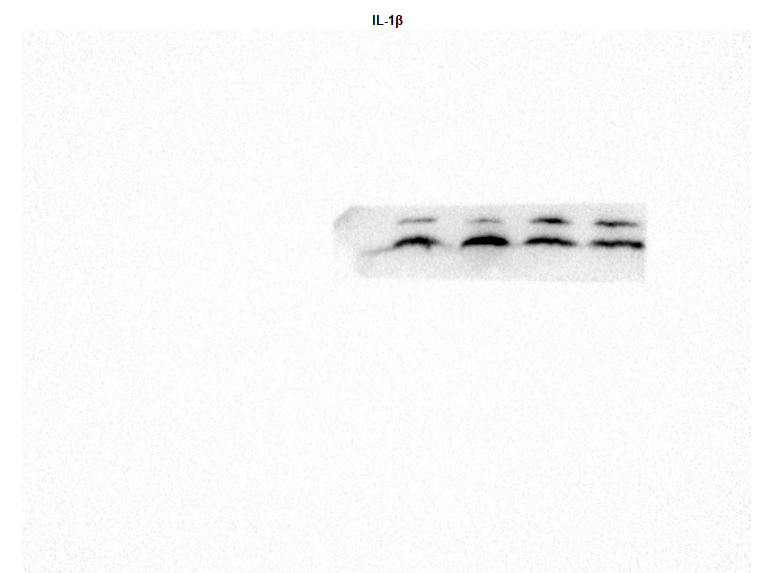

Supplement: Supplementary file 4 [file Data_Sheet_1.ZIP › IL-1β.tif]

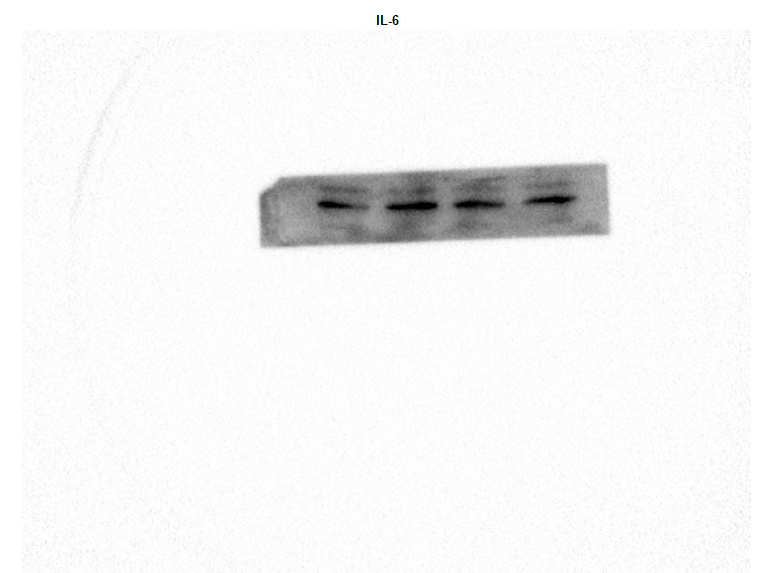

Supplement: Supplementary file 4 [file Data_Sheet_1.ZIP › IL-6.tif]

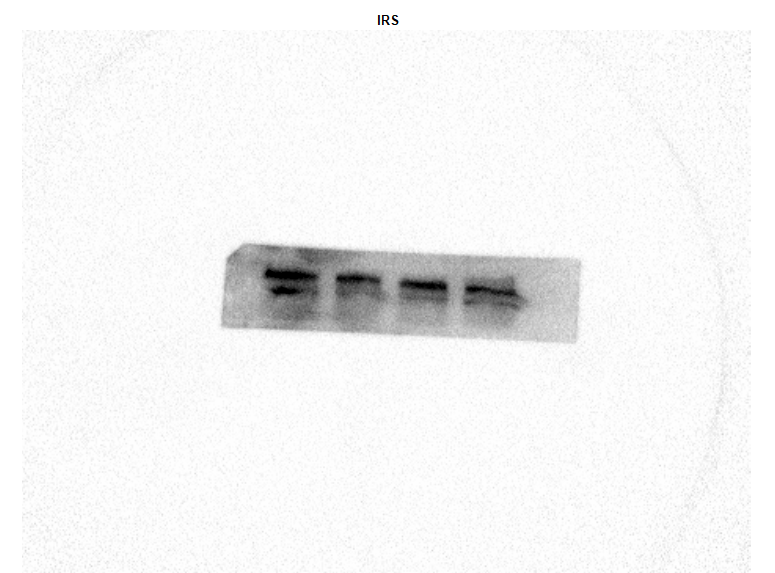

Supplement: Supplementary file 4 [file Data_Sheet_1.ZIP › IRS.tif]

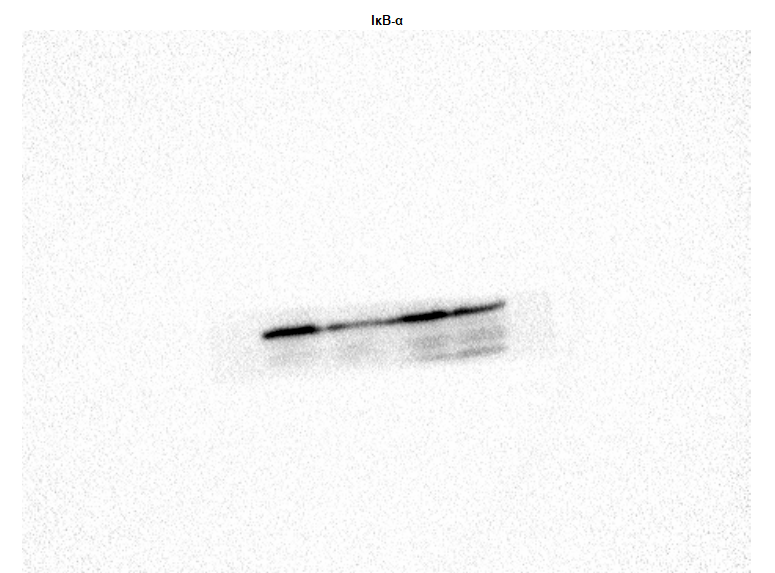

Supplement: Supplementary file 4 [file Data_Sheet_1.ZIP › IκB-α.tif]

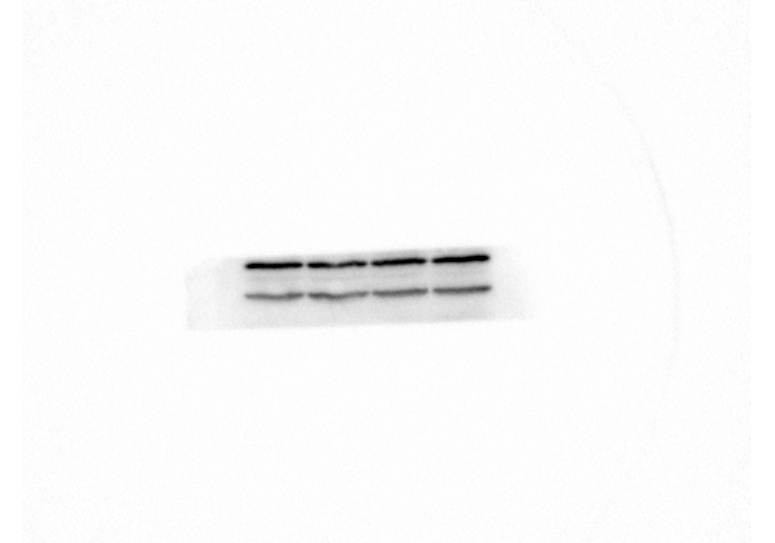

Supplement: Supplementary file 4 [file Data_Sheet_1.ZIP › JNK.tif]

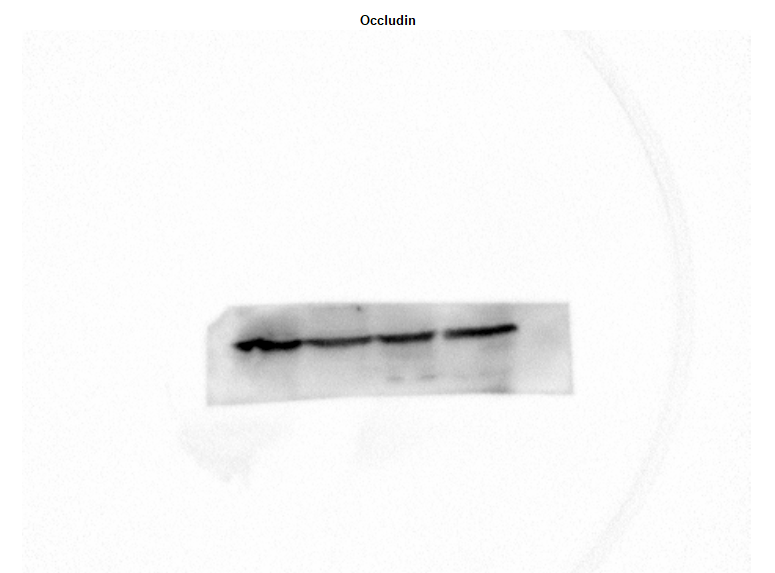

Supplement: Supplementary file 4 [file Data_Sheet_1.ZIP › Occludin.tif]

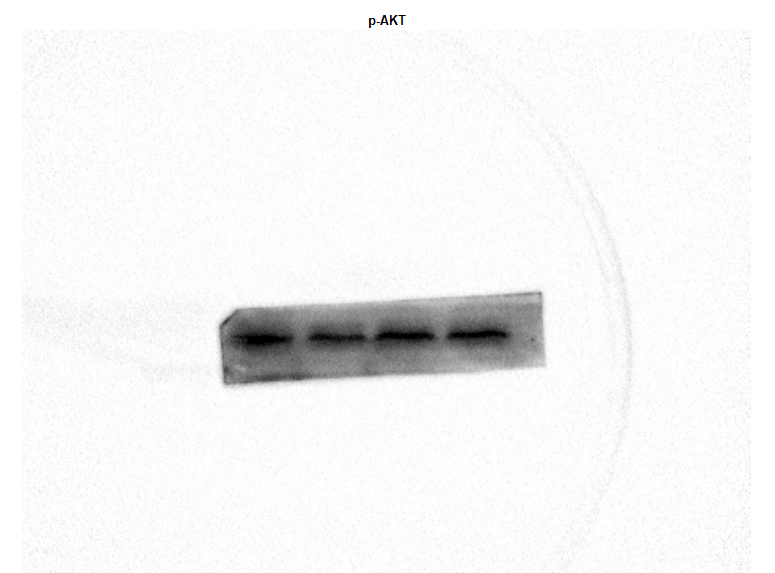

Supplement: Supplementary file 4 [file Data_Sheet_1.ZIP › p-AKT.tif]

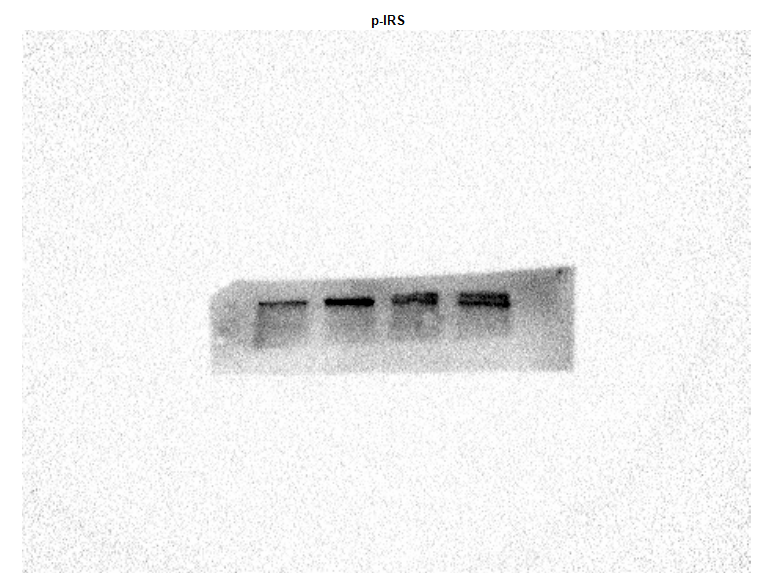

Supplement: Supplementary file 4 [file Data_Sheet_1.ZIP › p-IRS.tif]

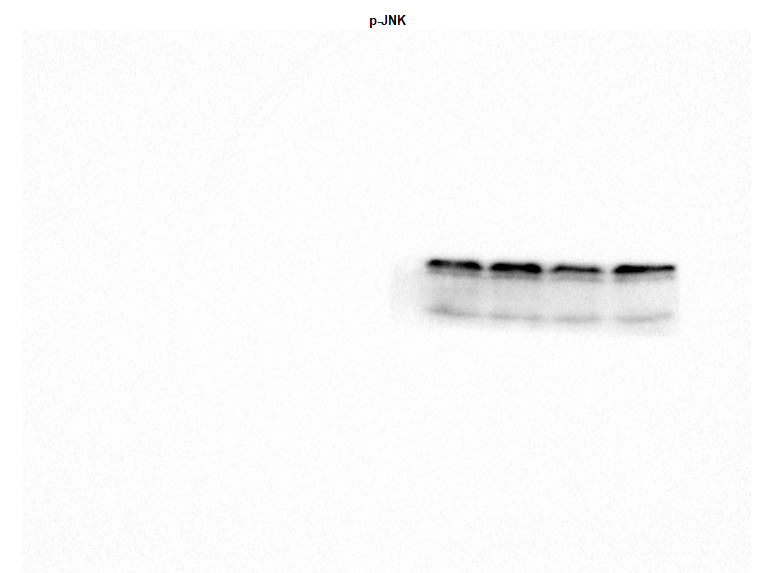

Supplement: Supplementary file 4 [file Data_Sheet_1.ZIP › p-JNK.tif]

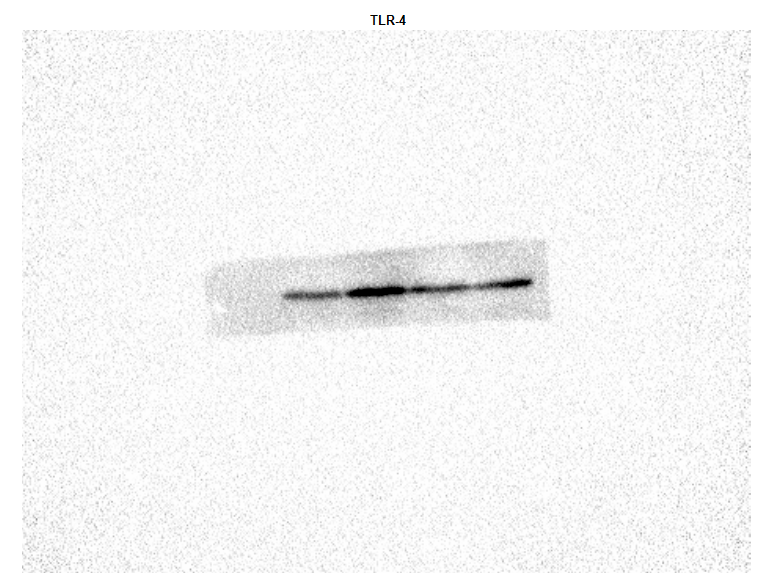

Supplement: Supplementary file 4 [file Data_Sheet_1.ZIP › TLR-4.tif]

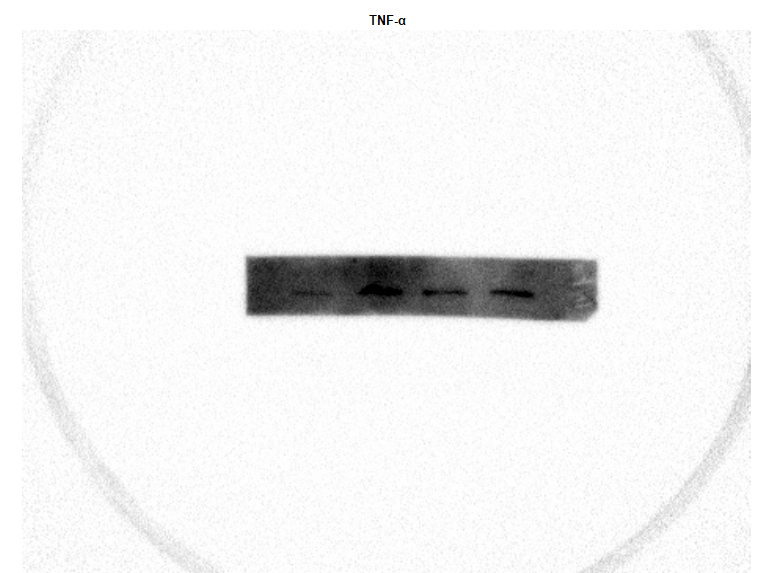

Supplement: Supplementary file 4 [file Data_Sheet_1.ZIP › TNF-α.tif]

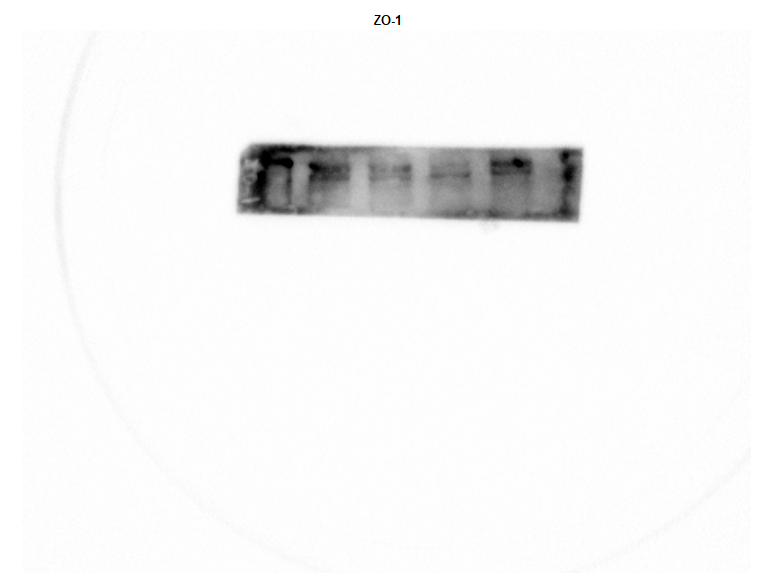

Supplement: Supplementary file 4 [file Data_Sheet_1.ZIP › ZO-1.tif]
